# Development of Biodegradable Polycation-Based Inhalable Dry Gene Powders by Spray Freeze Drying

**DOI:** 10.3390/pharmaceutics7030233

**Published:** 2015-08-26

**Authors:** Tomoyuki Okuda, Yumiko Suzuki, Yuko Kobayashi, Takehiko Ishii, Satoshi Uchida, Keiji Itaka, Kazunori Kataoka, Hirokazu Okamoto

**Affiliations:** 1 Faculty of Pharmacy, Meijo University, 150 Yagotoyama, Tempaku-ku, Nagoya 468-8503, Japan; E-Mails: tokuda@meijo-u.ac.jp (T.O.); 124331505@ccalumni.meijo-u.ac.jp (Y.S.); g0673221@ccalumni.meijo-u.ac.jp (Y.K.); 2 Department of Bioengineering, Graduate School of Engineering, The University of Tokyo, 7-3-1 Hongo, Bunkyo-Ku, Tokyo 113-8656, Japan; E-Mails: tishii@bmw.t.u-tokyo.ac.jp (T.I.); kataoka@bmw.t.u-tokyo.ac.jp (K.K.); 3 Division of Clinical Biotechnology, Center for Disease Biology and Integrative Medicine, Graduate School of Medicine, The University of Tokyo, 7-3-1 Hongo, Bunkyo-ku, Tokyo 113-8656, Japan; E-Mails: suchida@bmw.t.u-tokyo.ac.jp (S.U.); itaka-ort@umin.net (K.I.)

**Keywords:** dry powder inhalers (DPIs), pulmonary gene transfection, biodegradable polycations, spray freeze drying (SFD), porous particles

## Abstract

In this study, two types of biodegradable polycation (PAsp(DET) homopolymer and PEG-PAsp(DET) copolymer) were applied as vectors for inhalable dry gene powders prepared by spray freeze drying (SFD). The prepared dry gene powders had spherical and porous structures with a 5~10-μm diameter, and the integrity of plasmid DNA could be maintained during powder production. Furthermore, it was clarified that PEG-PAsp(DET)-based dry gene powder could more sufficiently maintain both the physicochemical properties and *in vitro* gene transfection efficiencies of polyplexes reconstituted after powder production than PAsp(DET)-based dry gene powder. From an *in vitro* inhalation study using an Andersen cascade impactor, it was demonstrated that the addition of l-leucine could markedly improve the inhalation performance of dry powders prepared by SFD. Following pulmonary delivery to mice, both PAsp(DET)- and PEG-PAsp(DET)-based dry gene powders could achieve higher gene transfection efficiencies in the lungs compared with a chitosan-based dry gene powder previously reported by us.

## 1. Introduction

A key for successful gene therapy is whether or not therapeutic genes can be effectively delivered and internalized into targeted organs and cells. Clinical trials for gene therapy against several intractable and lethal diseases have actively progressed in the world [1]. However, the approach for gene therapy by systemic administration has some obstacles, including degradation by endonuclease in blood and non-targeted distribution, subsequently leading to poor therapeutic effects and adverse effects, such as the interferon response. Therefore, the approach of local administration, which enables the direct delivery of therapeutic genes into target organs, has been examined [2].

Pulmonary administration is a powerful tool for achieving effective pulmonary gene therapy against several lung diseases, such as cystic fibrosis, α1-antitrypsin deficiency, and lung cancer, which are also the target of our pulmonary gene delivery system, due to direct and noninvasive delivery into deep lung areas through the respiratory tract [3]. Furthermore, viral and non-viral vectors have been applied to pulmonary gene delivery to realize higher gene transfection efficiencies [4]. On the other hand, the development of inhalable aerosol systems for pulmonary gene delivery is critical for clinical use. At present, three aerosol inhalation systems are available for clinical application: nebulizers, pressurized metered-dose inhalers (pMDIs), and dry powder inhalers (DPIs) [5]. A nebulizer formulation containing plasmid DNA (pDNA) encoding the therapeutic gene and a cationic liposome, as a vector, was used in a phase I clinical trial of gene therapy against cystic fibrosis [6,7].

Among these systems, DPIs have attracted much attention due to their low cost, the portable device, no propellant, and ease of handling [8]. Although reports on the development of DPIs for pulmonary gene therapy are still limited, some powder production techniques, including spray drying (SD), supercritical fluid precipitation, and lyophilization, might be applicable for a specific formulation or condition [9,10,11]. However, the destabilization of the gene or delivery system caused by several stresses, including heating, freezing, spraying, and shear force, must be considered during powder production [9,12,13,14,15]. Moreover, the morphology and particle size of prepared powders are critical factors affecting their inhalability. In general, aerodynamic particles of 1~5 μm are considered suitable for delivery by inhalation [8]. On the other hand, particles in this range of geometric size generate a strong adhesion force, resulting in poor dispersibility. To overcome the dilemma between aerodynamic and geometric particle sizes in the development of DPIs, recently, some low-density dry powders have been produced, facilitating an increase in the geometric particle size with a suitable aerodynamic particle size for inhalation [8].

Spray freeze drying (SFD) is a recent and simple technique to produce porous dry powders with a low density [16]. Maa *et al.* revealed that a dry powder produced by SFD could exhibit superior inhalation characteristics compared to that by SD irrespective of having similar geometric particle sizes [17]. In addition, SFD can guarantee the high-level recovery of produced dry powders, even if the initial amount of the formulation is small, which is important for the study of dry gene powders on a laboratory scale since the employed genes are relatively expensive. From these fine characteristics, we have introduced SFD into powder production for gene DPIs. In our previous study, it was demonstrated that dry gene powder could be stably prepared by SFD without the loss of pDNA integrity, and that the powder exhibited a gene expressing effect in the lungs of mice following pulmonary administration [18]. For clinical application, however, a higher gene transfection efficiency in the lungs is necessary.

The choice of suitable vectors for gene DPIs is a critical element determining gene transfection efficiency in the lungs following inhalation. Biodegradable polycations are attractive non-viral vectors for effective gene therapy because of their quick endosomal escape potential and high tolerability in the body [19]. In our previous reports, chitosan, a natural biodegradable polycation, was selected as a vector for inhalable dry gene powder prepared by SFD [18]. Chitosan has been reported to show high tolerability in the body [20]. Unfortunately, on the other hand, it has been reported that the transfection efficiency of chitosan is low compared to that of polyethyleneimine (PEI), a non-biodegradable polycation [21]. As novel biodegradable polycations for higher gene transfection efficiency, we have synthesized poly(aspartamide) derivatives with an ethylenediamine unit as a side chain (poly{*N*-[*N*-(2-aminoethyl)-2-aminoethyl]aspartamide} (PAsp(DET)) and their block copolymers with poly(ethylene)glycol (PEG-PAsp(DET)) [22,23]. These polycations have superior transfection efficiencies with minimal cytotoxicity compared to PEI, probably due to effective endosomal escape promoted by a marked shift of protonation between the physiological pH and endosomal acidic pH [24]. In particular, PEG-polycation block copolymers including PEG-PAsp(DET) can form a core-shell type of polyplex micelle composed of (1) the inner complex core constructed from electrostatic interaction between a negatively-charged gene and positively-charged segment in the polymers, and (2) the hydrophilic outer shell constructed from the PEG segment in the polymers, allowing it to escape the degradation of genes by endonuclease and aggregation caused by the interaction with blood components [25,26]. So far, PEG-PAsp(DET) polyplex micelles have successfully achieved the effective *in vivo* transfection of therapeutic genes into some target sites, such as: (1) vascular lesions via intraarterial administration [27], (2) a bone defect area in skull bone by regulated release from a calcium phosphate cement scaffold locally implanted [28], and (3) the lung via intratracheal administration [29]. These results prompted us to apply these polycations as vectors for effective gene DPIs with higher gene transfection efficiencies.

Thus, in this study, inhalable dry gene powders including PAsp(DET) and PEG-PAsp(DET) were produced by SFD. To improve the inhalable potential of dry powders prepared by SFD, l-leucine was added as a dispersibility enhancer in the formulation. The integrity of pDNA, physicochemical properties, and gene transfection efficiencies of the polyplexes after powder production were evaluated, respectively. Following the pulmonary administration of prepared dry powders to mice, the gene transfection efficiency and adverse effects in the lungs were compared to those of a chitosan-based dry powder developed in our previous study [18].

## 2. Experimental Section

### 2.1. Materials

The pDNA encoding firefly luciferase with CAG promoter (pCAG-Luc) was provided by RIKEN Bioresource Center (Tsukuba, Japan). pCAG-Luc was amplified in the DH5 strain of *Escherichia coli*, and purified using an EndoFree Plasmid Giga Kit (Qiagen GmbH, Hilden, Germany). The concentration and purity of pCAG-Luc were determined by measuring UV absorption at 260 nm and the A260/A280 ratio, respectively. d(−)-mannitol (Wako Pure Chemical Industries Ltd., Osaka, Japan) and l-leucine (Sigma–Aldrich, St. Louis, MO, USA) were used as an excipient and a dispersibility enhancer for inhalable dry powder, respectively. Chitosan (Mw: 2000~5000, water-soluble, Wako Pure Chemical Industries Ltd.) was used as a control vector in this study. Fluorescein sodium salt (FLNa; Sigma–Aldrich) and indocyanine green (ICG; Sigma–Aldrich) were selected as fluorescent labels of dry powders for *in vitro* inhalation and *in vivo* gene transfection studies, respectively. Luciferin (Promega, Madison, WI, USA) was used as a substrate of luciferase. Isoflurane (Abbott Laboratories, Abbott Park, IL, USA) was used for inhalation anesthesia in the measurement of luminescence and fluorescence in mice with an *in vivo* imaging system (IVIS^®^; IVIS-SPECTRUM, Caliper Life Sciences, Hopkinton, MA, USA). Lipopolysaccharide (Sigma–Aldrich) was selected as a positive control for evaluating lung injury *in vivo*. The other reagents and solvents used were of analytical grade.

### 2.2. Synthesis and Characterization of PAsp(DET) and PEG-PAsp(DET)

**Figure 1:**
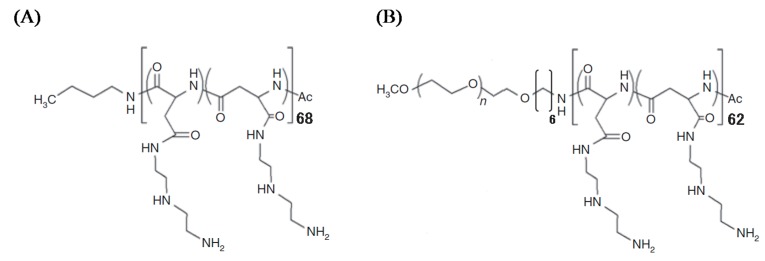
Chemical structures of (**A**) PAsp(DET) and (**B**) PEG-PAsp(DET).

PAsp(DET) and PEG-PAsp(DET) were prepared according to a ring-opening polymerization scheme, as previously reported [22,23]. Briefly, the polymerization of β-benzyl-l-aspartate *N*-carboxy-anhydride (Chuo Kaseihin Co., Inc., Tokyo, Japan) was started from *n*-butylamine (Wako Pure Chemical Industries Ltd.) for PAsp(DET) and α-methoxy-ω-amino PEG (Mw: ~12,000, Nippon Oil and Fats, Tokyo, Japan) for PEG-PAsp(DET) to obtain poly(β-benzyl-l-aspartate) (PBLA) and PEG-PBLA, respectively, followed by an aminolysis reaction to introduce diethylenetriamine (DET; Wako Pure Chemical Industries Ltd.) into the side chain of PBLA. From the ^1^H nuclear magnetic resonance spectrum, the polymerization degrees of PAsp(DET) and PAsp(DET) segments in PEG-PAsp(DET) were calculated to be 68 and 62, respectively, and the quantitative introduction of DET was confirmed (Figure 1).

### 2.3. Preparation of Dry Powders by SFD

**Figure 2:**
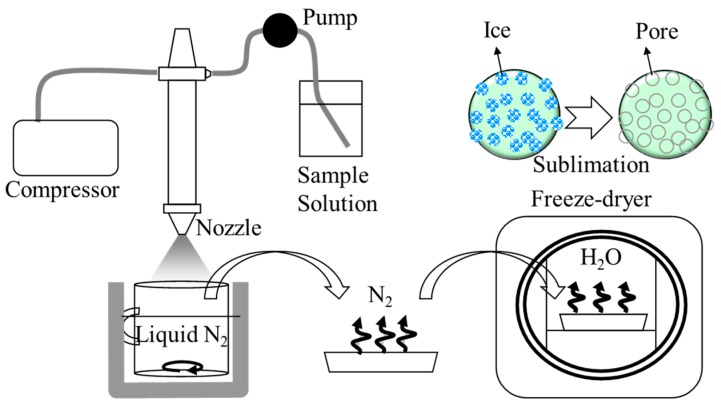
Diagram of the SFD setup.

The procedure of powder production by SFD was described in our previous report [18]. A schematic diagram is given in Figure 2. The formulations are listed in Table 1. The total mass of each formulation was adjusted to 50 mg. All formulation compositions for dry powders were dissolved in 2.5 mL of ultra-pure water. The solution was flowed at a rate of 5 mL/min, and atomized into liquid nitrogen at an air flow pressure of 150 kPa using a two-fluid nozzle (0.4 mm in inner diameter) for a spray dryer (SD-1000, TOKYO RIKAKIKAI Co., Ltd., Tokyo, Japan). We flowed water and air into the tubing connected with the nozzle before and after flowing the sample solution. The water flowed before the sample was to fix the flow rate and aid the wetting of the internal surfaces of the tubing and nozzle. The water flowed after the sample was to disperse the sample solutions left in the nozzle. The head of the nozzle was placed approximately 15 cm above the surface of liquid nitrogen. Following atomization, liquid nitrogen including frozen droplets was transferred to a freeze dryer (RLE-52, KYOWA VACUUM ENGINEERING Co., Tokyo, Japan) pre-cooled at a shelf temperature of −40 °C. After liquid nitrogen was evaporated, frozen droplets were dried at a pressure of less than 1 Pa, while the shelf temperature was gradually increased from −40 to 10 °C over a period of 24 h. The formulation names for prepared dry powders and solutions were abbreviated to “DP” and “SL” in each figure and table, respectively. The molecular ratio of amine in each polymer to phosphate in pDNA (N/P) was set to 4 or 8 for PAsp(DET), 40 or 80 for PEG-PAsp(DET), and 10 for chitosan, based on our previous studies [18,22,24,27,29].

Table 1Composition of (**A**) several dry powders prepared by SFD and (**B**) solutions (dosage/body). N/P: molecular ratio of amine in polymer to phosphate in pDNA, pDNA: plasmid DNA, Leu: l-leucine, ICG: indocyanine green, FLNa: fluorescein sodium salt, Man: d(−)-mannitol.

**Table:** (**A**) Dry powder

Formulation name	N/P	pDNA (μg)	Polymer (μg)	Leu (μg)	ICG or FLNa (μg)	Man (μg)	Total amount (mg)
Man DP	–	–	–	–	10	1490	1.5
Man-Leu DP	–	–	–	75	10	1415	1.5
PAsp(DET) DP	4 8	9 9	14.9 29.9	75 75	10 10	1391 1376	1.5 1.5
PEG-PAsp(DET) DP	40 80	9 9	255 510	75 75	10 10	1151 896	1.5 1.5
Chitosan DP	10	9	45	75	10	1361	1.5

**Table:** (**B**) Solution

Formulation name	N/P	pDNA (μg)	Polymer (μg)	Leu (μg)	ICG or FLNa (μg)	Man (μg)	Total amount (mg)	Water (μL)
PAsp(DET) SL	4	9	14.9	75	10	1391	1.5	50
PEG-PAsp(DET) SL	40	9	255	75	10	1151	1.5	50

### 2.4. Morphology and Particle Size of Dry Powders

To examine the morphology of prepared dry powders, the particles were observed using a scanning electron microscope (SEM, Type JSM-6060, JEOL Ltd., Tokyo, Japan). Before the observation, prepared dry powders were dispersed by an appropriate apparatus using pulmonary administration to mice [30]. The particle size distribution of the obtained powder was measured with the laser micron sizer LMS-30 (Seishin Enterprise Co., Ltd., Tokyo, Japan) based on laser diffraction.

### 2.5. Determination of pDNA Integrity in Dry Powders by Electrophoresis

Prepared dry powders were dissolved in ultra-pure water. We added 2 U of heparin sodium to enhance the dissociation of the complexes. Each sample solution containing 0.1 μg of pDNA was loaded on 0.6% agarose gel. Electrophoresis was carried out with a current of 100 V, 150 mA for 2 h in Tris–acetate–EDTA running buffer. A λ Hind III digest was used as a molecular mass marker, and naked pDNA and digested pDNA (by Hind III) were used as a control, respectively. After the electrophoresis, the gel was soaked in a 0.5 μg/mL ethidium bromide solution to detect pDNA with a fluorescent image analyzer (Typhoon FLA 9000; GE Healthcare Life Sciences, Tokyo, Japan).

### 2.6. Particle Size and Zeta Potential of Polyplexes

The particle size and zeta potential of the polyplexes formed between pDNA and PAsp(DET) or PEG-PAsp(DET) were assessed using an electrophoretic light scattering spectrophotometer (Zetasizer Nano ZS, Malvern Instruments, Worcestershire, UK) at 25 °C. Polyplex formation was performed in ultra-pure water, keeping it at room temperature for 30 min. Then, the final concentration of pDNA was adjusted to 20 μg/mL using 10 mM HEPES buffer at pH 7.4.

### 2.7. Cells

Murine colon adenocarcinoma (CT26) cells were kindly provided by Prof. Y. Takakura, Kyoto University. They were cultured in RPMI1640 medium supplemented with 10% heat-inactivated fetal bovine serum, 100 units/mL penicillin, and 100 μg/mL streptomycin at 37 °C in humidified air containing 5% CO_2_.

### 2.8. In Vitro Transfection Study by Polyplexes

CT26 cells were seeded on a 24-well microplate (Becton, Dickinson and Company, Franklin Lakes, NJ, USA) at a concentration of 60,000 cells/600 μL/well. After incubation for 24 h, the growth medium in each well was replaced with OPTI-MEM^®^ (Invitrogen Co., Carlsbad, CA, USA). Each polyplex was formed in ultra-pure water at room temperature for 30 min. Then, 60 μL of the polyplex solution was added to each well at an amount of 3 μg as pDNA. At 4 h after exposure, the OPTI-MEM^®^ containing the polyplex in each well was replaced with new growth medium, followed by incubation for 44 h. After removal of the medium, lysis buffer (0.05 % Triton X-100, 2 mM EDTA, 0.1 M Tris, pH 7.8) was added to each well to lyse cells. Additionally, each lysate was treated with three cycles of freezing and thawing to fully lyse cells. The lysate was centrifuged at 13,000× *g* for 7 min at 4 °C to collect the supernatant. The luciferase activity and protein concentration in each supernatant were measured by the Picagene^®^ luciferase assay (TOYO INK Co., Ltd., Tokyo, Japan) and Bradford protein assay, respectively.

### 2.9. In Vitro Inhalation Characteristics of Dry Powders

The *in vitro* inhalation characteristics of the prepared dry powders were evaluated using an 8-stage Andersen cascade impactor (AN-200; SIBATA SCIENTIFIC TECHNOLOGY Ltd., Saitama, Japan). To prevent dry powder from bouncing off the plates and becoming re-entrained in the air stream, the metal plates for the stages were coated with a thin layer of glycerin. After setting a No. 2 hydroxypropyl methylcellulose (HPMC) hard capsule (Shionogi Qualicaps Co. Ltd., Nara, Japan) containing 4 mg of each dry powder in a dry powder inhaler (Jethaler^®^ dual type; Hitachi Automotive Systems, Ltd., Isesaki, Japan), inspiration at a flow rate of 28.3 L/min and for a flow time of 5 s was carried out using a vacuum pump. After the inspiration, dry powder deposited in each part (capsule, device, throat, stages, and filter) was washed out by 10 mL of phosphate-buffered saline (PBS) to measure the concentration of FLNa using a fluorescence microplate reader (GEMINI EM; Molecular Devices LLC., Sunnyvale, CA, USA). From the concentration, the amount of dry powder deposited in each part was calculated. The deposition of dry powder in each part was defined by the following equation:

Deposition (%) = amounts in each part/amounts in total parts × 100(1)

For inhalation indexes, the output efficiency (OE) and fine particle fraction (FPF) were defined by the following equations, respectively:

OE (%) = amounts in throat and lower parts/amounts in total parts × 100(2)

FPF (%) = amounts in stage 3 and lower parts/amounts in throat and lower parts × 100(3)

The cut-off diameter in stage 3 was 4.7 μm. OE means the emission potential from the capsule and device, while FPF means the potential of delivery into the lung. The mass median aerodynamic diameter (MMAD) was determined from each deposition pattern, using special analysis software (AEROSOL particle density analysis system; SIBATA SCIENTIFIC TECHNOLOGY Ltd.).

### 2.10. Pulmonary Administration to Mice

All the animal experiments were approved by the Animal Care and Use Committee of Faculty of Pharmacy, Meijo University on 31 October 2011 (authorization number: Pharm-Exp-5) and conducted in accordance with the Guiding Principles for the Care and Use of Laboratory Animals. Female ICR mice weighing approximately 20 g were anesthetized with pentobarbital (50 mg/kg, i.p.) and a board was secured to their backs during the experiments. The trachea was exposed and 2.5 cm of PE-60 polyethylene tubing (internal diameter: 0.76 mm; Becton, Dickinson and Company, Franklin Lakes, NJ, USA) was inserted to a depth of 1.0 cm through an incision. Prepared dry powders were administered through the trachea using an appropriate apparatus for mice [30]. The powders (1.5 mg) were put in a disposable tip and dispersed in the trachea by releasing air (0.35 mL) compressed in a syringe by opening a three-way stopcock connecting the disposable tip and syringe. On the other hand, 50 µL of the solution dissolving 1.5 mg of a powder was similarly administered by a liquid aerosol apparatus for mice (MicroSprayer^®^-Model IA-1C; Penn-Century Inc., Philadelphia, PA, USA).

### 2.11. Detection of Fluorescence and Luminescence in Vivo

Following the pulmonary administration of each formulation to mice, fluorescence and luminescence in mice were detected using IVIS^®^ as in our previous report, respectively [18,31]. The nominal pixel size and minimum detectable radiance of IVIS^®^ were 13.5 μm and 70 photons/s/cm^2^/sr, respectively. To detect the fluorescence derived from ICG, an excitation filter of 745 nm and an emission filter of 820 nm were used, and the exposure time was set to 1 s. When the luminescence corresponding to luciferase activity was detected, luciferin as a substrate of luciferase was administered (150 mg/kg, i.p.) at 10 min before the detection point, and the exposure time was set to 1 min. During the measurement, the mice were anesthetized with isoflurane on a stage kept at 25 °C. To quantify the fluorescence and luminescence intensities in the lungs of mice, the region of interest (ROI) was adjusted to a rectangle with a width of 3 cm and height of 1 cm.

### 2.12. Histological Analysis of Excised Lung

For this evaluation, mice were selected with sufficient pulmonary gene expression in the *in vivo* gene transfection study described above. At 48 h following the pulmonary administration of each formulation to mice, cardiac perfusion from left the ventricle to right atrium with PBS was performed to wash out the blood in the lungs. Then, the lungs were fixed by perfusion with 4% paraformaldehyde in PBS. The fixed lungs were excised from the bodies and stored in 4% paraformaldehyde in PBS at 4 °C for 24 h. Subsequently, they were transferred into 30% sucrose in PBS and stored at 4 °C for 48 h to fully remove paraformaldehyde. Cryosections (10 μm) were cut from the lungs frozen in O.C.T. Compound (Sakura Finetek USA, Inc., Torrance, CA, USA) on dry ice, and stained by hematoxylin & eosin to observe them with a microscope (BIOREVO BZ-9000; KEYENCE, Osaka, Japan).

### 2.13. Statistical Analysis

Statistical comparisons were made using Aspin-Welch’s *t*-test for evaluation of the particle size, zeta potential, and *in vitro* transfection efficiencies of polyplexes formed before and after powder production, and a one-way ANOVA followed by Dunnett’s test for the other evaluations. Significant correlations between pulmonary delivery and gene expression *in vivo* were evaluated based on a test of no correlation. *P* < 0.05 was considered significant.

## 3. Results

### 3.1. Morphology and Particle Size of Dry Gene Powders Prepared by SFD

**Figure 3:**
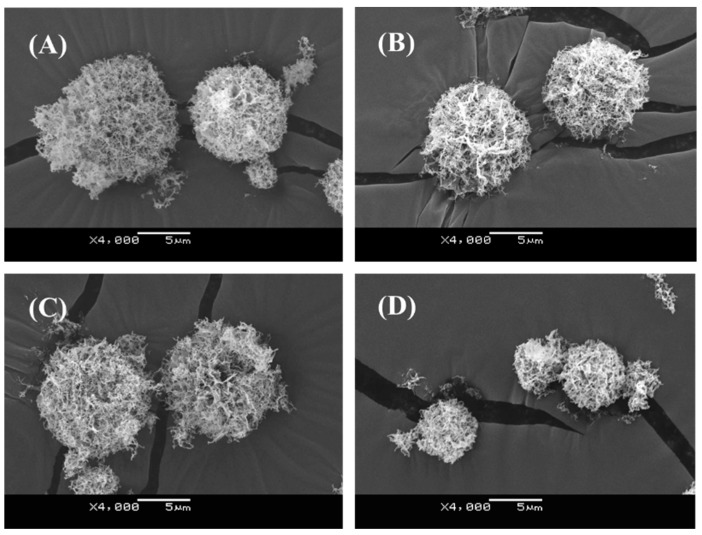
Scanning electron micrographs of (**A**) Man DP, (**B**) Man-Leu DP, (**C**) PAsp(DET) DP (N/P = 4), and (**D**) PEG-PAsp(DET) DP (N/P = 40).

All dry powders could successfully be collected by SFD. The recovery of dry powders was achieved at a rate of more than 60% of the theoretical amount. From Figure 3 and Table 2, it was confirmed that the dry powders had spherical and porous shapes with a diameter of approximately 10 μm. Morphological and size differences were not observed between dry powders of different compositions.

**Table 2:** Geometric particle sizes of Man DP, Man-Leu DP, PAsp(DET) DP (N/P = 4), and PEG-PAsp(DET) DP (N/P = 40). Each value represents the mean ± S.D. (*n* = 3). Significant difference compared with Man DP (*, *P* < 0.05).

Formulation name	Cumulative particle size (μm)		
	*d*_10_	*d*_50_	*d*_90_
Man DP	4.4 ± 0.7	8.5 ± 1.0	19.0 ± 3.9
Man-Leu DP	4.6 ± 0.4	10.3 ± 0.5	27.7 ± 3.7
PAsp(DET) DP	3.7 ± 0.2	7.6 ± 0.3	14.4 ± 0.4
PEG-PAsp(DET) DP	4.7 ± 0.3	11.7 ± 1.8 *	28.4 ± 6.6

### 3.2. Stability of pDNA during Powder Production by SFD

**Figure 4:**
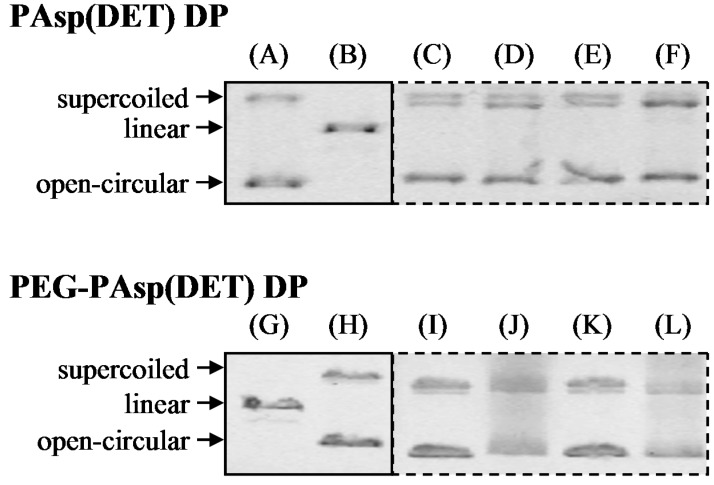
Integrity of pDNA in PAsp(DET) DP and PEG-PAsp(DET) DP after powder production by SFD. (**A**,**H**) naked pDNA, (**B**,**G**) pDNA digested with Hind III, (**C**,**D**) PAsp(DET) polyplex before and after powder production of PAsp(DET) DP (N/P = 4), (**E**,**F**) PAsp(DET) polyplex before and after powder production of PAsp(DET) DP (N/P = 8), (**I**,**J**) PEG-PAsp(DET) polyplex before and after powder production of PEG-PAsp(DET) DP (N/P = 40), and (**K**,**L**) PEG-PAsp(DET) polyplex before and after powder production of PEG-PAsp(DET) DP (N/P = 80). Electrophoresis of the samples for PAsp(DET) DP and PEG-PAsp(DET) DP was separately carried out with the individual gels. In the fluorescent image of each gel, the regions related to this study (shown as solid and dotted lines) were selectively cut off to combine with no modification. The position of each band in the combined images is the same as that in the original ones.

To evaluate the integrity of pDNA in the prepared dry gene powders, electrophoresis was carried out. As controls, two bands derived from naked pDNA represent the supercoiled and open-circular forms (Figure 4A,H), while that from digested pDNA represents the linear form (Figure 4B,G). In all dry gene powders prepared at different N/P ratios, bands corresponding to the supercoiled and open-circular forms of pDNA could be detected, but that corresponding to the linear form could not (Figure 4D,F,J,L), being similar to the results for sample solutions before powder production by SFD (Figure 4C,E,I,K). These results indicate that the integrity of pDNA could be maintained during powder production by SFD.

### 3.3. Physicochemical Properties and In Vitro Gene Transfection Efficiencies of PAsp(DET) and PEG-PAsp(DET) Polyplexes Reconstituted from Dry Gene Powders Prepared by SFD

**Figure 5:**
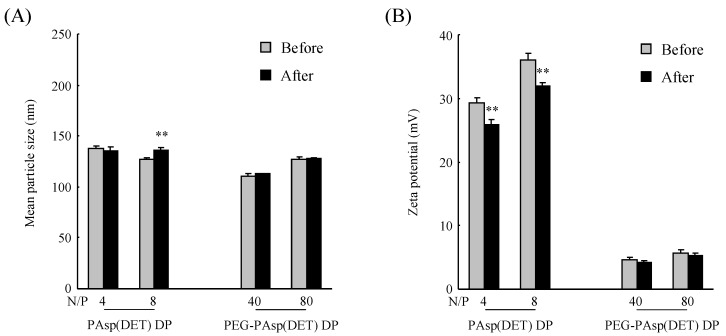
(**A**) Particle size and (**B**) zeta potential of polyplexes formed before and after powder production by SFD. Each powder was dissolved in ultra-pure water to reconstitute the polyplex. Each value represents the mean ± S.D. (*n* = 3). Significant difference compared with before power production (**, *P* < 0.01).

Physicochemical properties of a polyplex including the particle size and surface charge are important factors for determining its cellular binding and uptake potentials [32]. Therefore, the particle size and zeta potential of PAsp(DET) and PEG-PAsp(DET) polyplexes reconstituted from dry gene powders were measured and compared with those before powder production (Figure 5). It was clarified that the particle size and zeta potential of PAsp(DET) and PEG-PAsp(DET) polyplexes were not markedly changed after powder production, suggesting preservation of the physicochemical properties of these polyplexes during powder production by SFD. The mean particle sizes of PAsp(DET) and PEG-PAsp(DET) polyplexes ranged from 100 to 150 nm. The zeta potential of the PAsp(DET) polyplex was more positive at a higher N/P, while that of the PEG-PAsp(DET) polyplex remained neutral irrespective of N/P.

**Figure 6:**
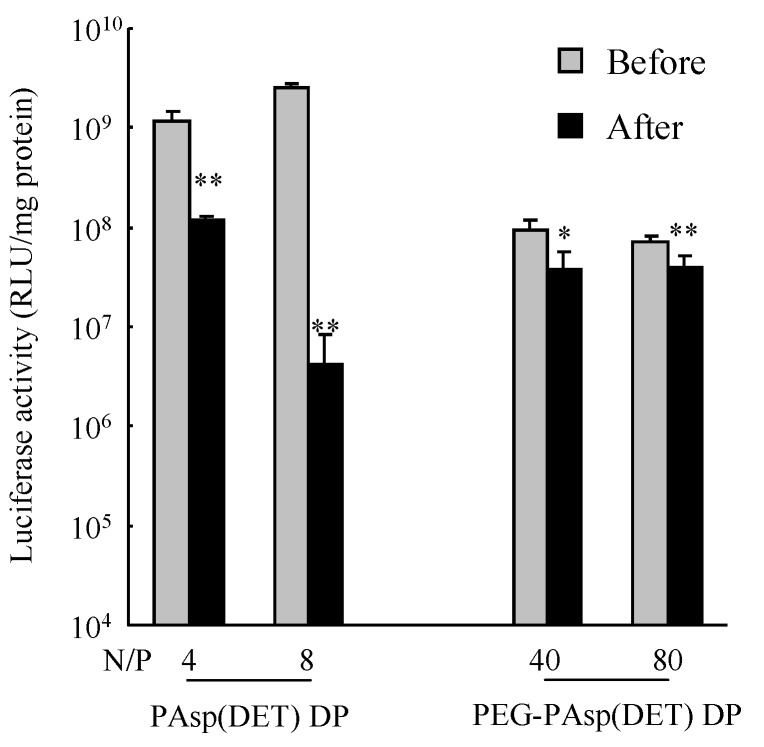
*In vitro* gene transfection efficiencies against CT26 cells by polyplexes formed before and after powder production by SFD. Each powder was dissolved in ultra-pure water to reconstitute the polyplex. Each value represents the mean ± S.D. (*n* = 4). Significant difference compared with before powder production (**, *P* < 0.01; *, *P* < 0.05).

Next, the *in vitro* gene transfection efficiencies of PAsp(DET) and PEG-PAsp(DET) polyplexes reconstituted from dry gene powders were evaluated and compared with those before powder production (Figure 6). Before powder production, it was confirmed that the PAsp(DET) polyplex had a more than 10-fold higher gene transfection efficiency compared with the PEG-PAsp(DET) polyplex. After powder production, however, the efficiencies of PAsp(DET) polyplexes at N/P of 4 and 8 decreased to approximately 9.8% and 0.17% of those before powder production, respectively, indicating the marked losses of their activities during powder production. On the other hand, the efficiencies of PEG-PAsp(DET) polyplexes at N/P of 40 and 80 after powder production were approximately 40% and 55% of those before powder production, respectively, suggesting the higher stability of PEG-PAsp(DET) polyplexes for transfection efficiency in powder production.

### 3.4. Inhalation Characteristics of Dry Gene Powders Prepared by SFD

**Figure 7:**
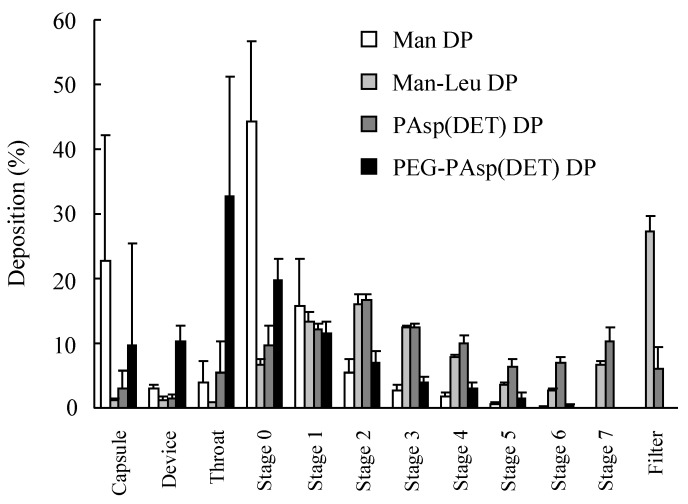
*In vitro* deposition patterns of Man DP, Man-Leu DP, PAsp(DET) DP (N/P = 4), and PEG-PAsp(DET) DP (N/P = 40) in an 8-stage Andersen cascade impactor following inspiration at a flow rate of 28.3 L/min and for a flow time of 5 s. Each value represents the mean ± S.D. (*n* = 3).

For evaluating the inhalable potential of prepared dry powders, an 8-stage Andersen cascade impactor was used. Following inspiration, most of the Man DP was deposited in the capsule and stage 0, while a larger amount of Man-Leu DP was deposited in stage 3 and lower parts (Figure 7). From a comparison of inhalation indexes for Man DP and Man-Leu DP (Table 3), it was clarified that the addition of l-leucine markedly increased the value of OE from 74% up to 98% and the value of FPF from 6.8% up to 62%, respectively, indicating the enhanced dispersibility and lung deposition potentials. Regarding dry gene powders, the values of OE and FPF in PAsp(DET) DP were 96% and 54%, respectively, which were nearly equal to those in Man-Leu DP. On the other hand, the values of OE and FPF in PEG-PAsp(DET) DP were 80% and 11%, respectively, which were similar to those in Man DP in spite of the addition of l-leucine. MMADs in Man-Leu DP and PAsp(DET) DP were approximately 1~3 μm, being a suitable aerodynamic diameter for lung delivery by DPIs.

**Table 3:** Several inhalation indexes of Man DP, Man-Leu DP, PAsp(DET) DP (N/P = 4), and PEG-PAsp(DET) DP (N/P = 40), calculated from *in vitro* deposition patterns. OE: output efficiency, FPF: fine particle fraction, MMAD: mass median aerodynamic diameter. Each value represents the mean ± S.D. (*n* = 3). Significant difference compared with Man DP (**, *P* < 0.01).

Formulation	OE (%)	FPF (%)	MMAD (μm)
Man DP	74.3 ± 19.2	6.8 ± 1.3	15.6 ± 2.5
Man-Leu DP	97.8 ± 0.9	62.3 ± 3.0 **	1.2 ± 0.2 **
PAsp(DET) DP	95.5 ± 2.9	54.3 ± 6.2 **	2.4 ± 0.5 **
PEG-PAsp(DET) DP	79.9 ± 13.2	11.2 ± 1.6	8.7 ± 1.5 **

### 3.5. In Vivo Pulmonary Gene Transfection Efficiencies of Dry Gene Powders Prepared by SFD

To perform *in vivo* studies of inhalable dry powders including pharmacokinetic and pharmacological studies, in general, the powders are dispersively administered into the lungs of small animals by intratracheal insufflation using some specific apparatuses [30,33]. However, the spontaneous breathing of small animals disturbs the exact dose control of the powders deposited in the lungs, consequently leading to unclear results in *in vivo* studies. To estimate the *in vivo* transfection efficiencies by inhalable dry gene powders more precisely, in our previous reports, we clarified the usefulness of a noninvasive dual imaging system for evaluating both pulmonary delivery and gene expression by inhalable dry gene powders in each mouse [18,31]. Fluorescence derived from ICG and luminescence corresponding to luciferase activity in each mouse represent lung delivery and gene expression by a dry gene powder, respectively. In our previous reports, a significant correlation between fluorescence and luminescence intensities in mice was demonstrated. Furthermore, a slope of the regression line gained from the correlation diagram shows the *in vivo* pulmonary gene transfection efficiency of a dry gene powder, compensated for the scattering of pulmonary delivery in mice.

**Figure 8:**
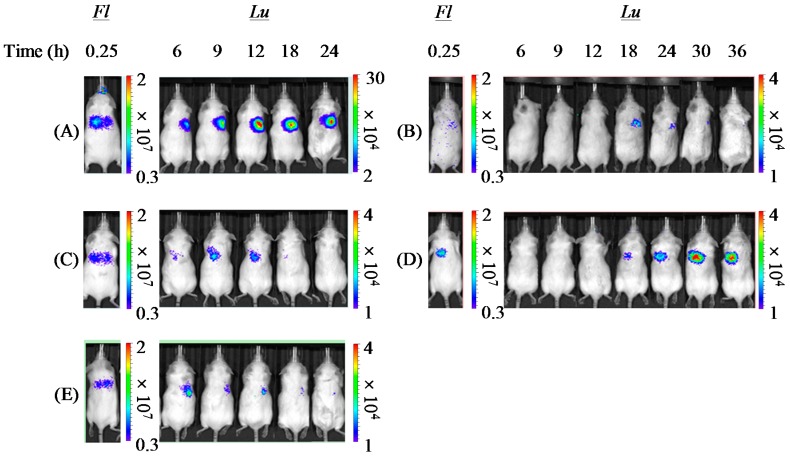
Optical images of pulmonary delivery and gene expression by (**A**) PAsp(DET) DP (N/P = 4), (**B**) PEG-PAsp(DET) DP (N/P = 40), (**C**) PAsp(DET) SL (N/P = 4), (**D**) PEG-PAsp(DET) SL (N/P = 40), and (**E**) Chitosan DP (N/P = 10). The pulmonary delivery and gene expression were evaluated by the detection of fluorescence derived from ICG (*Fl*) and luminescence corresponding to luciferase activity (*Lu*) using IVIS^®^ following pulmonary administration to mice, respectively. The color scales are in photons/s/cm^2^/sr.

**Figure 9:**
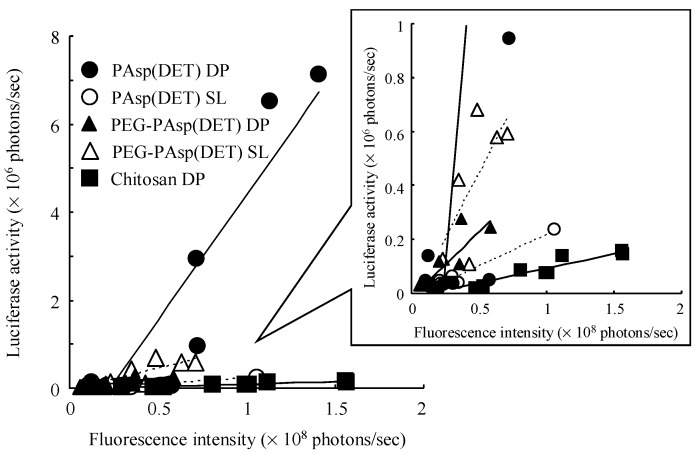
Correlation between pulmonary delivery and gene expression by PAsp(DET) DP and SL (N/P = 4), PEG-PAsp(DET) DP and SL (N/P = 40), and Chitosan DP. In the correlation diagram, fluorescence intensity derived from ICG at 15 min and maximum luminescence intensity corresponding to luciferase activity, which were calculated by IVIS^®^, following pulmonary administration into mice were plotted, respectively. The calculated intensities were corrected using each pre-administered mouse.

Using this system, in the present study, the *in vivo* pulmonary gene transfection efficiencies of novel dry gene powders (PAsp(DET) DP and PEG-PAsp(DET) DP) were compared with the solution formulations (PAsp(DET) SL and PEG-PAsp(DET) SL) and a chitosan-based dry gene powder (Chitosan DP) that we developed previously [18]. In PAsp(DET) DP, PAsp(DET) SL, and Chitosan DP, the maximum luminescence intensities were detected at 6~12 h following pulmonary delivery to mice, while these were at 18~36 h in PEG-PAsp(DET) DP and PEG-PAsp(DET) SL (Figure 8). In Figure 9, the correlation between the fluorescence intensity derived from ICG at 15 min and the maximum luminescence intensity corresponding to luciferase activity in each mouse following pulmonary administration was plotted. A significant correlation was observed in each formulation except PEG-PAsp(DET) SL. By comparison between the slopes of the regression lines, it was demonstrated that all formulations with PAsp(DET) and PEG-PAsp(DET) polyplexes had higher *in vivo* pulmonary gene transfection efficiencies than Chitosan DP (Table 4). Among them, PAsp(DET) DP had the highest transfection efficiency, being approximately 50-fold higher than Chitosan DP.

**Table 4:** Indexes for regression lines determined from a correlation diagram between pulmonary delivery and gene expression by PAsp(DET) DP and SL (N/P = 4), PEG-PAsp(DET) DP and SL (N/P = 40), and Chitosan DP. Significant correlation between pulmonary delivery and gene expression was evaluated from a test of no correlation (**, *P* < 0.01; *, *P* < 0.05).

Formulation name	*n*	*r*^2^	Slope
PAsp(DET) DP	9	0.842 **	568
PAsp(DET) SL	9	0.959 **	23
PEG-PAsp(DET) DP	9	0.719 *	49
PEG-PAsp(DET) SL	6	0.501	98
Chitosan DP	9	0.861 **	11

### 3.6. In Vivo Toxicities of Dry Gene Powders Prepared by SFD

**Figure 10:**
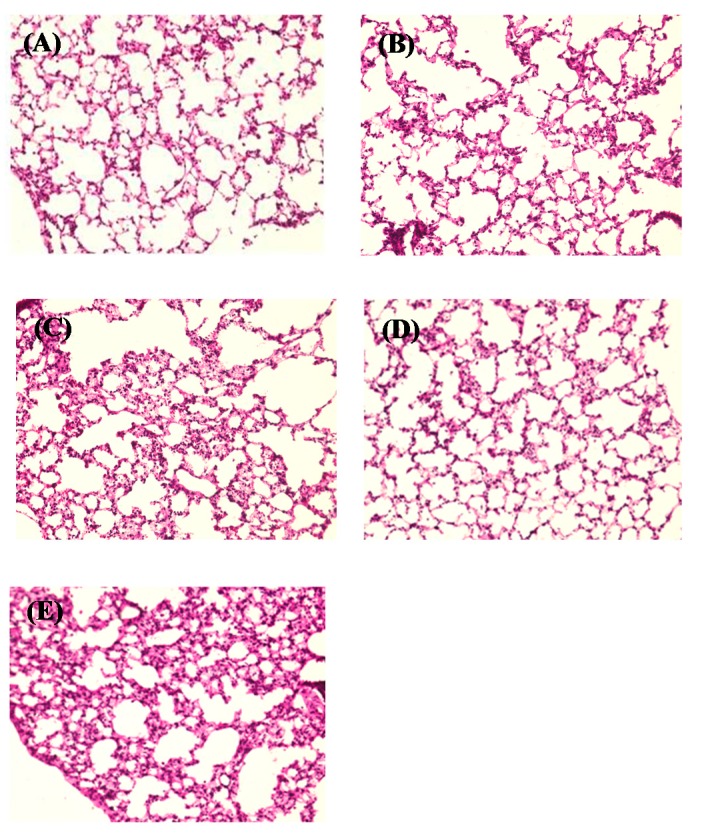
Histological observation of lungs excised at 48 h following the pulmonary delivery of (**C**) PAsp(DET) DP (N/P = 4) and (**D**) PEG-PAsp(DET) DP (N/P = 40) into mice. (**A**) Water and (**B**) Man-Leu DP were administered as negative controls, and (**E**) lipopolysaccharide (120 mg/kg) was a positive control.

To assess the toxicities of dry gene powders in the lungs, histological observations were performed using mice with sufficient pulmonary gene expression in the *in vivo* gene transfection study described above. The alveolar structure of mice treated with PEG-PAsp(DET) DP (Figure 10D) remained intact as did those treated with water (Figure 10A) and Man-Leu DP (Figure 10B), negative controls, suggesting the possibility of its mild irritation. On the other hand, an increase in the filtration of inflammatory cells was observed in the alveolar structure of mice treated with PAsp(DET) DP (Figure 10C), although it was not as marked as that of mice treated with lipopolysaccharide (Figure 10E), a positive control inducing acute lung inflammation [34]. Therefore, these results indicate that PAsp(DET) DP could cause more marked irritation than PEG-PAsp(DET) DP.

## 4. Discussion

A chitosan-based dry gene powder prepared by SFD in our previous report [18] was not sufficient for human clinical application as an inhalant because of the relatively large particle size (approximately 20~40 μm). In this study, therefore, the atomizing pressure in the preparation procedure was increased from 20 to 150 kPa, consequently achieving the production of smaller-sized dry powders (Figure 1 and Table 2). Even in the powder production with more marked atomizing stress, the integrity of pDNA in PAsp(DET) DP and PEG-PAsp(DET) DP was demonstrated to be maintained (Figure 4). Similar results were observed in some gene powder productions containing cationic vectors [9,15]. Thus, the polyplex formation between an anionic gene and a cationic vector in a sample solution for powder production must markedly contribute to avoiding the critical loss caused by some stresses in the procedure of SFD.

In our previous report, we used mouse lung metastasis models, which were prepared by the intravenous injection of CT-26 cells into mice, to examine the *in vivo* efficacy of a chitosan-interferon-β gene complex powder for inhalation [35]. In the present study, we thus selected CT26 cells in the *in vitro* gene transfection study, expecting a future *in vivo* efficacy study with the same mouse lung metastasis models. The decreased *in vitro* gene transfection efficiency of the PAsp(DET) polyplex after powder production (Figure 6) was considered to be caused by the increased binding affinity of the DET segment with pDNA in the PAsp(DET) polyplex. Ramsy *et al.* suggested that the binding affinity of a cationic poly(amino acid) vector with a gene is a more essential factor in determining the gene transfection efficiency of the polyplex than its particle size and surface charge [36]. Moreover, Hahn *et al.* reported that the binding affinity of PEI, a cationic vector, with pDNA was increased in a PEI polyplex reconstituted after powder production by lyophilization [37]. The increased binding affinity interfered with the dissociation of pDNA from the PEI polyplex in the cytosol, consequently leading to a lower gene transfection efficiency. In this study, therefore, a similar phenomenon would occur in the PAsp(DET) polyplex reconstituted from PAsp(DET) DP. On the other hand, the maintained *in vitro* gene transfection efficiency of the PEG-PAsp(DET) polyplex after powder production (Figure 6) could be explained by the role of the PEG segment in PEG-PAsp(DET). In sample solution for powder production, PEG-PAsp(DET) formed the core-shell type of polyplex micelles, as mentioned above, which would maintain the environment of the inner complex core during powder production to maintain the binding affinity of the DET segment with pDNA in polyplex micelles. Armstrong *et al.* reported that PEG modification of a cationic liposome could maintain the *in vitro* gene transfection efficiency of the lipoplex reconstituted after powder production by lyophilization, partly supporting our hypothesis [38].

l-Leucine has been widely used as a dispersant reagent for inhalable dry powders prepared by SD [39,40]. Although the mechanism under enhanced dispersibility by l-leucine has not been fully clarified, l-leucine spontaneously covers the surface of droplets after aerosolization of sample solution due to a surfactant-like property and low solubility in water or other vehicles, which might contribute to lowering the cohesiveness and adhesiveness of prepared dry powders [41]. In this study, therefore, l-leucine was applied to powder production by SFD to further promote inhalation performance. The tap density of Man-Leu DP was 0.0094 g/mL, which was much lower than that of Man DP, 0.035 g/mL. The surface areas of these two particles measured by argon gas adsorption were 26.6 and 17.8 m^2^, respectively, suggesting that these particles were porous. The water content measured with the Karl Fischer method was 0.89% and 0.86%, respectively. In the prepared dry gene powders, PAsp(DET) DP showed favorable inhalation performance similar to Man-Leu DP, while PEG-PAsp(DET) DP did not in spite of the addition of l-leucine (Figure 7 and Table 3). This result could be easily explained by the difference of polymer occupation in the composition (PAsp(DET) DP; 1%, PEG-PAsp(DET); 17%). In our previous studies, it was clarified that PEG-PAsp(DET) needed larger amounts (*i.e.*, higher N/P) than PAsp(DET) to show effective gene expression, because the outer PEG shell would hamper the cellular uptake and intracellular process of the polyplex [27]. Thus, a high N/P was similarly chosen for PEG-PAsp(DET) DP in this study. However, greater polymer occupation in PEG-PAsp(DET) DP would markedly affect its inhalation performance. This observation strongly indicated that we must select a vector for an effective inhalable dry gene powder in view of not only its gene transfection efficiency but also effective inhalability.

From the slope of the regression line gained from the correlation diagram between pulmonary delivery and gene expression by each formulation (Table 4), it was demonstrated that both PAsp(DET) DP and PEG-PAsp(DET) DP had higher *in vivo* pulmonary gene transfection efficiencies compared with Chitosan DP, which was in accordance with the higher *in vitro* gene transfection potentials of these polymers themselves. In the *in vitro* gene transfection study (before powder production), as shown in Figure 6, both PAsp(DET) and PEG-PAsp(DET) polyplexes showed superior gene-expressing effects (1~10 × 10^8^ RLU/mg protein), while the chitosan polyplex did not (<1 × 10^4^ RLU/mg protein, data not shown). These results suggest that a vector with a higher gene transfection potential should be selected for developing an effective dry gene powder for inhalation.

The much higher *in vivo* gene transfection efficiency of PAsp(DET) DP than PAsp(DET) SL (Table 4), which was consistent with our previous report about a chitosan-based dry gene powder prepared by supercritical CO_2_ precipitation [10], surprised us since it was opposite to the results of the *in vitro* gene transfection study (Figure 6). Although the reasons for this discrepancy between *in vivo* and *in vitro* remain unclear, one of them is considered to be the different concentration of the polyplex exposed on the cell surface. On reaching the lungs following pulmonary delivery *in vivo*, PAsp(DET) DP was dissolved in a small volume of water on the lung epithelial surface to reconstitute a very high concentration of the PAsp(DET) polyplex. The high-concentration exposure might enhance the intracellular uptake of the PAsp(DET) polyplex, contributing to the higher *in vivo* gene transfection efficiency of PAsp(DET) DP. On the other hand, the difference between *in vivo* gene transfection efficiencies of PEG-PAsp(DET) DP and PEG-PAsp(DET) SL (Table 4) were in good agreement with the results of the *in vitro* gene transfection study (Figure 6). It has been reported that PEG modification of lipoplexes and polyplexes interferes with the electrostatic interaction between positively charged complexes and the negatively charged cell membrane, resulting in limited intracellular uptake via endocytosis [42,43]. Possibly, the intracellular uptake of the PEG-PAsp(DET) polyplex might be restricted even at an extremely high concentration of polyplex formation on the lung epithelial surface, consequently leading to similar trends between *in vitro* and *in vivo* gene transfection efficiencies of PEG-PAsp(DET) DP and SL. In addition, the PEG segment in PEG-PAsp(DET) must also be related to the delayed gene expression of PEG-PAsp(DET) DP and SL (time of maximum gene expression: 6~12 h in PAsp(DET) DP and SL, 18~36 h in PEG-PAsp(DET) DP and SL), which was consistent with the results of the transfection study using multicellular tumor spheroids, as shown in a previous report [44]. From the comparison between *in vitro* and *in vivo* gene transfection efficiencies, it was strongly indicated that the *in vivo* gene transfection efficiency of an intact dry gene powder cannot be effectively predicted based on the *in vitro* gene transfection efficiency of its dissolved form.

Although we did not record the emitted dose in this study, it was the case that some powders remained in the tip. Even if all the powder was emitted from the tip, the powder distribution in the lung would vary among animals. To overcome this problem, we employed the dual imaging technique [18,31]. PAsp(DET) DP showed the highest *in vivo* gene transfection efficiency and higher FPF than PEG-PAsp(DET) DP. However, the *in vitro* dispersion was conducted with 4 mg powder at 28.3 L/min from Jethaler^®^. On the other hand, the *in vivo* dispersion was conducted with 1.5 mg powder from a syringe device. The dispersion conditions and device design were different, and so the results cannot be directly compared. For clinical applications, an Andersen cascade impactor study with an appropriate device containing an optimal amount of powder is required.

Local toxic actions induced by some lipoplexes and polyplexes on the lung epithelial surface have limited clinical application as gene inhalation therapy [45]. In human clinical trials, it was reported that the nebulization of a lipoplex containing GL67, a cationic lipid optimized for pulmonary gene therapy, caused some influenza-like symptoms (myalgia, headache, and a high body temperature) and mild respiratory symptoms (cough, wheeze, and a tight chest) [6,7]. Therefore, a guarantee of safety in the lungs is essential for the clinical application of gene formulations for inhalation. The present study indicated that PEG-PAsp(DET) DP had good tolerability but PAsp(DET) DP could cause more severe irritation in the lungs (Figure 10). Similar trends were observed in our previous reports with their solution formulations [29,46], partly supporting the present results. The cationic surface charge is one of the most important factors related to the toxicities of lipoplexes and polyplexes, which can cause direct membrane damage and aggregation in the body. After the dissolution of PEG-PAsp(DET) DP on the lung epithelial surface, the PEG-PAsp(DET) polyplex would be reconstituted, in which the hydrophilic PEG shell covers the inner cationic core, greatly contributing to its favorable tolerability. Our preliminary study with a pulse oximeter (MouseOx^®^, STARR Life Sciences, Oakmont, PA, USA) showed no changes in the oxygen saturation, number of breaths, and heartbeat rate of mice 24 and 48 h following the pulmonary administration of each formulation (9 μg as pDNA), suggesting no severe toxic effect of PAsp(DET) and PEG-PAsp(DET) polyplexes in solution and powder (data not shown). Unfortunately, information about the safety of PAsp(DET) DP and PEG-PAsp(DET) DP is limited in the present report, and comprehensive analysis including quantifications of some indicators (e.g., lactate dehydrogenase activity and inflammatory cytokine amounts in bronchoalveolar fluid) will be necessary to confirm their local and systemic safety more precisely. As a future plan to achieve a higher transfection efficiency in the lungs with minimized toxicity, we will try to apply the combination of PAsp(DET) and PEG-PAsp(DET) to the powder formulation for inhalation, whose efficiency was shown in our previous report with the solution formulation [46].

In addition to the local toxicity of the gene complex, that of the carrier material is important. Lactose is commonly used as an additive for inhalable formulations; however, it is usual to use lactose as a carrier which is not expected to be delivered deep into the lungs. In the present study, mannitol was used as a carrier delivered deep into the lungs with the gene complex. Mannitol has been used as a carrier for SFD powders in published reports [47,48]. The *in vivo* toxicity study showed that Man-Leu was not toxic (Figure 10); however, the selection of a carrier is one of the important issues to be resolved in the future.

## 5. Conclusions

In this study, novel biodegradable polycation (PAsp(DET) and PEG-PAsp(DET) polymers)-based inhalable dry gene powders could be stably prepared by SFD. It was clarified that the prepared dry powders had porous shapes with an approximately 10-μm diameter, and that the addition of l-leucine could markedly improve the inhalation characteristics of the powders. Moreover, powder production by SFD could maintain the physicochemical properties of PAsp(DET) and PEG-PAsp(DET) polyplexes, but could not do so with the *in vitro* gene transfection efficiencies. On the other hand, it was demonstrated that PAsp(DET)- and PEG-PAsp(DET)-based dry gene powders had higher *in vivo* gene transfection efficiencies than a chitosan-based dry gene powder which we developed previously. As for the safety of PAsp(DET) DP and PEG-PAsp(DET) DP in the lungs, it was indicated that PEG-PAsp(DET) DP showed favorable tolerability but PAsp(DET) DP could cause more marked irritation. These results suggest that both PAsp(DET)- and PEG-PAsp(DET)-based dry gene powders prepared by SFD might be good candidates for efficient pulmonary gene therapy.
